# Coffee consumption and periodontitis: a Mendelian Randomization study

**DOI:** 10.1186/s12263-023-00732-3

**Published:** 2023-09-09

**Authors:** Wan-Zhe Liao, Zhi-Yi Zhou, Zi-Kai Lin, Shuo-Jia Xie, Ya-Fang Zheng, Jun-Tao Wang, Jun-Huang Zheng, Hao-Kai Chen, Wu-Shu Chen, Xu-Guang Guo

**Affiliations:** 1 Department of Clinical Laboratory Medicine, Guangdong Provincial Key Laboratory of Major Obstetric Diseases, Guangdong Provincial Clinical Research Center for Obstetrics and Gynecology, The Third Affiliated Hospital of Guangzhou Medical University, Guangzhou, 510150 China; 2 Department of Clinical Medicine, The Nanshan College of Guangzhou Medical University, Guangzhou, 511436 China; 3 Department of Clinical Medicine, The Third Clinical School of Guangzhou Medical University, Guangzhou, 511436 China; 4 Department of Clinical Medicine, The Fourth Clinical School of Nanchang University, Nanchang, 330031 China; 5 Guangdong Provincial Key Laboratory of Major Obstetric Diseases, The Third Affiliated Hospital of Guangzhou Medical University, Guangzhou, 510150 China; 6 Guangzhou Key Laboratory for Clinical Rapid Diagnosis and Early Warning of Infectious Diseases, KingMed School of Laboratory Medicine, Guangzhou Medical University, Guangzhou, China

**Keywords:** Coffee consumption, Periodontitis, Bidirectional Mendelian Randomization, Periodontal health

## Abstract

**Background:**

Coffee is one of the most consumed beverages in the world, coffee consumption has been growing in the United States over the past 20 years. Periodontitis is defined by the pathologic loss of the periodontal ligament and destruction of the connective tissue attachment and alveolar bone loss and is related to different systemic diseases and conditions. However, the causality has remained unclarified, thus we regarded discovering the causal relationship between coffee consumption and the liability to periodontitis as the objective of the study.

**Methods:**

Coffee consumption was subdivided into binary coffee consumption and continuous coffee consumption to refine the study design. Genetic instruments were stretched from the MRC-IEU’s (MRC Integrative Epidemiology Unit) output from the GWAS pipeline using phesant-derived variables based on the UK Biobank, the Gene-Lifestyle Interactions in Dental Endpoints (GLIDE) project, and the joint meta-analysis of a recent GWAS. The IVW (Inverse Variance Weighted) was regarded as the primary method to estimate the causality, a scatter plot revealed the intuitive result, and tests for stability were also carried out.

**Results:**

An effect of continuous coffee consumption on the risk of periodontitis was found, with per SD of coffee consumed increases, the risk of periodontitis rises by 1.04% (Odds Ratio of IVW is 1.0104), while the effect of binary coffee consumption on periodontitis did not meet the requirement of indicating a strong causal association, neither were the reverse causality analyses.

**Conclusions:**

The study indicated the causality of continuous coffee consumption to the risk of periodontitis with a relatively small scale of effect estimate and no strong evidence for an effect of binary coffee-consuming behavior on periodontitis. There was also no intensive evidence suggesting reverse causality.

**Supplementary Information:**

The online version contains supplementary material available at 10.1186/s12263-023-00732-3.

## Introduction

Coffee is one of the most consumed beverages in the world, with an estimated 2.25 billion cups drunk worldwide per day [1–3]. Over the past 20 years, coffee consumption in the United States has been growing, nowadays, about two-thirds of American adults drink coffee every day [4]. Because of the widespread popularity of coffee, the fact that the potential benefits or risks related to coffee intake may have a greater impact on public health has drawn scientists’ significant attention, with the relationship between coffee consumption and oral diseases particularly concerned [5–7].

Oral diseases affect approximately half of the global population, with severe periodontitis being one of the most prevalent non-communicable diseases which was also an under-acknowledged and important public health problem, the eleventh most prevalent global disease in 2016 [8–10]. Periodontitis is defined by the pathologic loss of the periodontal ligament and alveolar bone and is the inflammation of the periodontium that leads to the destruction of the connective tissue attachment and alveolar bone loss [11, 12]. Periodontitis has been potentially suggested to be related to many various systemic diseases and conditions, and it is not only a local phenomenon but also related to the overall health status [13]. A meta-analysis shows a trend in association between periodontitis and cognitive impairment[14]. A study about the systemic effects of the treatment for periodontitis in patients diagnosed with T2DM (type 2 diabetes mellitus) suggested that periodic oral health assessment and treatment of periodontitis could be of importance for the effective management of type 2 diabetes [15]. The infection of periodontitis is also considered to adverse pregnancy outcomes [16, 17].

There were few studies have discussed the association between coffee and periodontitis [8, 13, 18, 19]. Nevertheless, the causal relationship between coffee consumption and periodontitis is not clear and has not been established among these observational studies due to methodological defects. Compared with conventional methods, Mendelian Randomization (MR), which increasingly appeared in epidemiological studies, has the capacity of linking the proposed risk factor and outcome and estimating the causal effect with less confounding and bias through the difference in the outcome between individuals carrying the genetic variant and ones do not [20, 21]. In the nutshell, the study discovered the impact of coffee intake on the infection of periodontitis using MR.

## Materials and methods

### Overview of study design and population

**Fig. 1:**
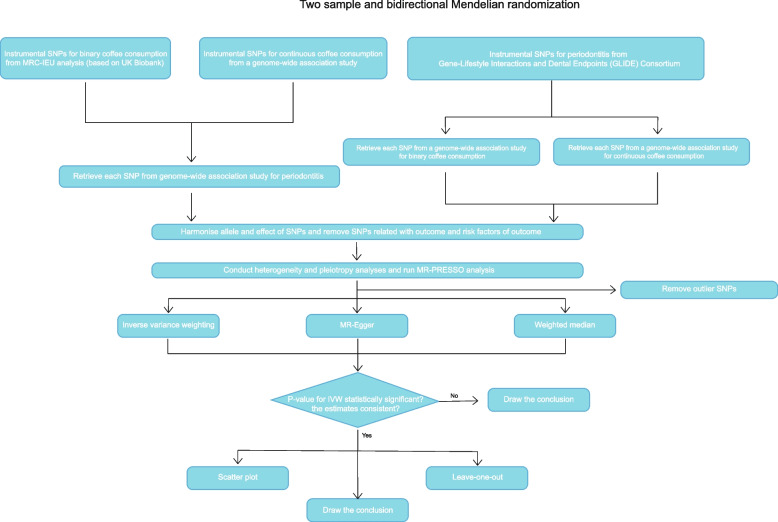
Flow chart of the MR-based study revealing causality from coffee consumption on periodontitis. SNP, single nucleotide polymorphism; MRC-IEU, MRC Integrative Epidemiology Unit; IVW, inverse variance weighted; MR-PRESSO, MR Pleiotropy RESidual Sum and Outlier

A detailed overview of the study was shown in Fig. 1. For the meticulousness of the research, we divided the variable “coffee consumption” into two parts: the binary coffee consumption (“Did you drink coffee yesterday”) and continuous intake of coffee (“How many cups of coffee do you drink each day” (include decaffeinated coffee)) among participants. The process of screening, exclusion, and other statistical analysis was based on the R package “TwoSampleMR”, which was constructed by Gibran Hemani et al. All participants enrolled in the study were limited to Europeans. GWAS summary statistics data from GLIDE and MRC-IEU analysis that based on UK Biobank were attached in the Supplementary File 5 and 6.

### Selection of instrumental variables for coffee consumption

To get accurate IVs, SNPs were identified for binary coffee intake directly from MRC-IEU analysis of UK Biobank phenotypes. According to the three key consumptions during the MR analysis process: 1. The IVs are strongly associated with the exposure variable; 2. The IVs only affect the outcome via affecting the exposure; 3. The IVs are stipulated not to be related to any confounders of the exposure-outcome relationship [22, 23]. The genome-wide significance threshold for screening SNPs associated with exposure interest was set as *P* < 5e-8 while the standard for linkage disequilibrium (LD) among SNPs for exposure was defined by r2 > 0.001 and clump distance < 10000 kb. Only the “leader” with the least *P*-value was contained when multiple SNPs were confirmed at the same locus. To satisfy the assumptions that IVs are not supposed to be related to outcome or any confounding factors, after SNPs were all retrieved from GWAS summary data of outcome, every SNP was searched at the ieu open gwas project website (https://gwas.mrcieu.ac.uk) to examine for signs of pleiotropy and whether strongly associated confounding factors or periodontitis, the outcome (*P* < 5e-8), then SNPs related to outcome or at least two risk factors (including smoking, alcohol consumption, metabolic syndrome, hyperlipidemia, and diabetes mellitus) for periodontitis were removed [24, 25] (Raw SNPs and raw results in the Raw analysis without removing any SNPs strongly related to outcome or more than two risk factors were attached in Supplementary Table 4, along with the reasons for SNPs removing in the primary analysis). The F-statistic for every SNP was calculated to avoid weak instrument bias, the left ones (F > 10) were the IVs expected. As for IVs for continuous coffee consumption, we identified SNPs from a recent GWAS (Genome-Wide Association Study) based on data from UK Biobank, Nurses’ Health Study, Health Professionals Follow-up Study, and Women’s Genome Health Study (*N* = 375833) [26], which represented a more convincing instrumentality than direct extracting from UK Biobank. Then, IVs were obtained after the steps above.

### Selection of instrumental variables for periodontitis

With the objective of proceeding with the implementation of bidirectional Mendelian Randomization, IVs for periodontitis are stretched from the Gene-Lifestyle Interactions in Dental Endpoints (GLIDE) project [27, 28] and candidate SNPs were screened for validity meeting the three key assumptions described above. We particularly chose the Autosomal and EUR versions of single-trait GLIDE summary statistics data (*N* = 49066, 17672 cases versus 31394 controls) to avoid latent overlap on participants from UK Biobank while targeted participants were still limited to Europeans. Similarly, IVs for periodontitis were reached through the steps above except for the calculation of the F-statistics due to the deficiency of effect allele frequencies. while the genome-wide significance threshold was amplified to 5e-6 due to all SNPs being relatively weakly associated with the risk of periodontitis.

### Bidirectional Mendelian Randomization between coffee consumption and periodontitis

In MR of coffee consumption on periodontitis, GWAS of Summary level for periodontitis and coffee consumption were obtained from GLIDE consortium by Shungin et al., and IEU analysis of UK Biobank phenotypes [27–29]. IVs of binary (*N* = 64949, 45788 cases versus 19161 controls) and continuous coffee intake (*N* = 428800) associated with periodontitis were stretched respectively. Then the process of harmonization was carried out to harmonize the alleles and effects between the exposure and outcome: correcting strands for non-palindromic SNPs and dropping all palindromic SNPs from the study. The SNPs filtered were waiting for further statistical analyses for MR estimates.

Concerning the bias caused by unidirectional Mendelian randomization, and secondary causal confounding effects, we decided to do reverse MR to construct an intact bidirectional analysis. As for MR of periodontitis on coffee consumption, we retrieve the summary statistics data of binary and continuous coffee consumption straightly from MRC-IEU analysis of UK Biobank phenotypes. After information from coffee consumption and periodontitis for every single nucleotide was combined, we also conducted the harmonization.

### Heterogeneity and pleiotropy analyses

For the final filtration of the four separated MR, Cochran’s Q test and MR-Egger intercept test were introduced to examine the potential heterogeneity and pleiotropy of the combined SNPs. Only when *P*-values for Cochran’s Q and Egger intercept were all beyond 0.05 further MR estimates analyses for the SNPs would be carried out, otherwise, MR-PRESSO would be conducted to specify and exclude the outlier SNPs.

### Analyses of MR estimates

**Fig. 2:**
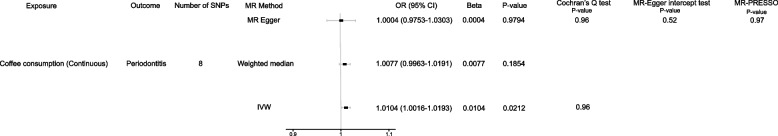
Odds ratio plot for the effect of continuous coffee consumption on periodontitis. OR, odds ratio; MR-PRESSO, MR Pleiotropy RESidual Sum and Outlier

**Fig. 3:**
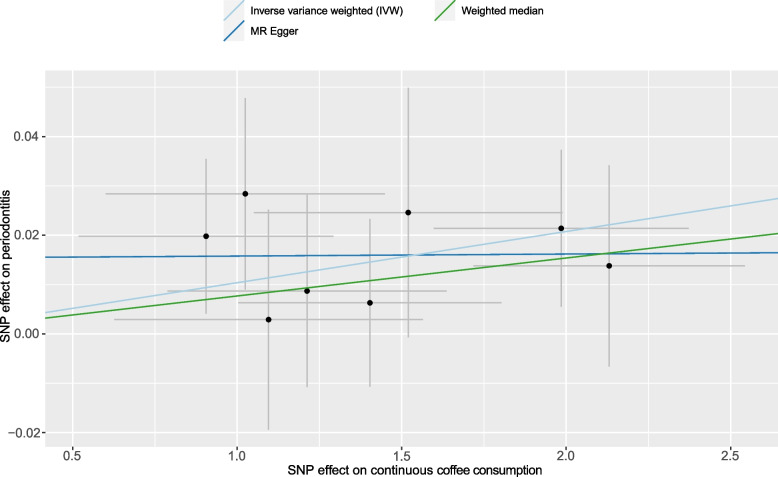
Scatter plot for the effect of continuous coffee consumption on periodontitis through genetic variants

**Fig. 4:**
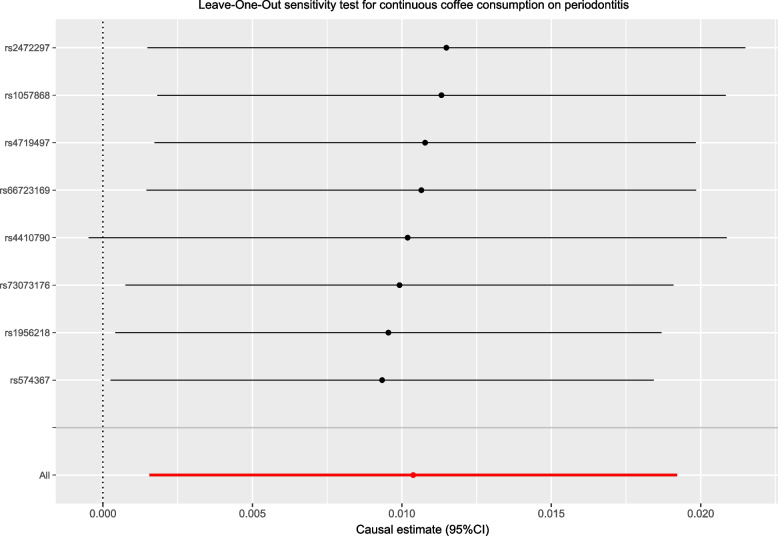
Leave-One-Out plot for sensitivity test for the effect of continuous coffee consumption on periodontitis

In the part of MR, estimates of IVW (Inverse Variance Weighted) were regarded as the main criterion for judging the result, and outcomes of MR-Egger and Weighted median were used for the validation of general plus or minus directions of the effect from exposure on the outcome, as MR-Egger and Weighted median could provide more robust result in a loose set of scenarios, despite being less efficient [30, 31]. If the estimates were consistent and IVW’s result was statistically significant, a Scatter figure would be plotted and Leave-One-Out analyses would be conducted to give impetus to finalize the sensitivity analysis. Otherwise, if the estimates were inconsistent or IVW’s result was not significant, the conclusions would be drawn: there is no specific causality between assumed exposure and outcome. Correspondences between exposure and outcome and the characteristics of the SNP were revealed in Fig. 2, Table 1, S1, S2, S3, and scatter plot and Leave-One-Out plot for the causality between continuous coffee consumption and periodontitis that passed the statistical inspection were shown in Figs. 3 and 4.

**Table 1:** Characteristics of the SNPs used for the causality from continuous coffee consumption on periodontitis. SNP, single nucleotide polymorphism; EAF, effect allele frequency; SE, standard error

			Continuous coffee consumption (Exposure)					Periodontitis (Outcome)				
SNP	Effect Allele	Other Allele	EAF	Beta	SE	*P*-value	Sample size	EAF	Beta	SE	*P*-value	Sample size
rs1057868	T	C	0.29	1.4036	0.4	5.26E-33	375,833	NA	0.0063	0.017	0.71	49,066
rs1956218	G	A	0.56	0.9055	0.3873	3.62E-08		NA	0.0198	0.0157	0.21	
rs2472297	T	C	0.27	2.1307	0.4123	5.19E-155		NA	0.0138	0.0204	0.50	
rs4410790	C	T	0.63	1.9849	0.3873	5.59E-141		NA	0.0214	0.0159	0.18	
rs4719497	T	C	0.87	1.0954	0.4690	4.23E-08		NA	0.0029	0.0223	0.90	
rs574367	T	G	0.21	1.0247	0.4243	8.06E-09		NA	0.0284	0.0194	0.14	
rs66723169	A	C	0.23	1.2124	0.4243	9.88E-17		NA	0.0087	0.0195	0.66	
rs73073176	C	T	0.87	1.5199	0.4690	5.56E-25		NA	0.0246	0.0253	0.33

## Result

### Causality from coffee to periodontitis

Based on the 8 SNPs that remained after removing the outlier ones which have strong correlations with the outcome factor, we found intensive evidence of the latent causal effect between binary coffee consumption and the risk of periodontitis using the inverse variance weighted model: OR = 1.0104, 95%CI: 1.0016–1.0193, *P* = 0.0212, which indicated that with per SD of coffee consumed, the risk of periodontitis rises by 1.04%. Despite *P* values for MR-Egger regression and weighted median model were not deemed forceful evidence for statistical significance, the estimates were all consistent with IVW: OR = 1.0004, 95%CI: 0.9753–1.0303, *P* = 0.98 for MR-Egger regression, and OR = 1.0077, 95%CI: 0.9963–1.0191, *P* = 0.19 for the weighted median model. Heterogeneity and horizontal pleiotropy were not observed utilizing Cochran’s Q-test (P_MR-Egger_ = 0.9582, P_IVW_ = 0.9605) and MR-Egger intercept test (*P* = 0.5192) with MR-PRESSO test was conducted for further validation (*P* = 0.97) (Fig. 2). The Leave-One-Out plot and Scatter plot were shown in Figs. 3 and 4. We noticed that the left terminus of the confidence interval of rs4410790 exceeded the invalid line in the Leave-One-Out test, based on the previous heterogeneity and pleiotropy tests, the small number of SNPs was considered to be the reason. By contraries, it illustrated the outstanding effect of rs4410790 on the casualty from binary coffee consumption to the risk of periodontitis.

Intriguingly, as for the causal effect of continuous coffee intake on the liability of periodontitis, little evidence suggested a statistically significant association of genetically predicted periodontitis with the binary consumption of coffee in Table S1: Beta = 0.2210, Se = 0.3574, *P* = 0.54.

### Causality from periodontitis to coffee consumption

Heading for the objective of eliminating the bias caused by retrocausality, causality tests were conducted previously. Utilizing the 6 Periodontitis-related SNPs, we found no significant results sustaining the latent reverse causation whether binary coffee consumption or continuous coffee consumption served as the outcome in IVW method: OR = 0.9988, 95%CI: 0.9856–1.0122, *P* = 0.86 for the binary, and Beta = -0.0016, Se = 0.0048, *P* = 0.73 for the continuous (Table S2, S3), with heterogeneity and pleiotropy tested negative. Indeed, no obvious causal effect was observed from the risk of periodontitis to coffee consumption.

## Discussion

The authors implemented multiple MR approaches to address the possible causal association between coffee intake and infection risk of periodontitis. To the best of our knowledge, up to now, the causal relationship between coffee intake and periodontitis has remained unclarified. Though a previous study reported the antimicrobial activity and the protective effect on *Porphyromonas gingivalis,* one of the risk indicators of periodontitis, of chlorogenic acid which works as an essential ingredient of coffee [32]. Nevertheless, there is no evidence for the accurate correlation between coffee consumption according to Yeonjae Rhee et. al’s meta-analysis based on existing 2 cohort studies and 4 cross-sectional studies focusing on the association, while the conventional design (cases versus controls), reverse effect and ambiguous temporal sequence still existed [13]. For the objective of avoiding the influence of confounding risk factors and reverse causality, the study was carried forward through bidirectional Mendelian randomization and the specified definition of binary and continuous coffee consumption.

In line with the above findings, the MR-based study indicated continuous coffee intake had a causality on the risk of periodontitis, with no evidence suggesting the causal effect of binary coffee consumption on periodontitis. Reverse causality was also not found.

According to the extant studies, it is foreseeable that coffee consumption has a prospective effect on the progression of periodontitis. Coffee contains a variety of chemical compositions, caffeine (1,3,7-trimethylxanthine) is one of the main alkaloid components in coffee fruits and the source of the bitter taste of coffee [13]. Kamagata-Kiyoura, Y et al. reported that the possible implication of routine caffeine intake in the acceleration of pathological conditions of periodontitis [33]. A study in rats that evaluated the function of caffeine suggested that Caffeine increased bone loss in ligated teeth [34]. Dal-Fabbro et al. reported that excessive caffeine intake increases bone resorption associated with periapical periodontitis in rats, and the inflammatory pattern deriving from periapical periodontitis alters the expression of RANKL, IL-1β, and TRAP [35]. Not come singly but in pairs, another research for rats also demonstrated that daily intake of high doses of caffeine may enhance ligature-induced periodontitis progression [36]. However, as another major component of coffee, chlorogenic acid plays a divergent role on periodontitis by exerting anti-inflammatory properties [37]. A recent study indicated that chlorogenic acid could attenuate inflammation in Human gingival fibroblasts [38]. Li, Han and his colleagues found that bone loss in mouse periodontitis could be alleviated by sustained release of chlorogenic acid-loaded nanomicelles [39]. Therefore, we regard the differentiated effects of coffee ingredients as an explanation for the relatively weak effect size of continuous coffee consumption, and no strong association was found between whether consume coffee and the infection of periodontitis. On the contrary, Song Jianan et al. found that the combination of caffeine and chlorogenic acid attenuated LPS-induced PGE2 release and enhanced the anti-oxidative effect, prompting potential modification still to be discovered [40].

We acknowledge that the study was equipped with some strengths. To begin with, Mendelian Randomization endowed us with the capacity of discovering causality by simulating randomized control trials in observational settings. With a high level of confidence, MR avoids the input of substantial time cost, funding, and confounding bias for SNPs, which were assigned at conception randomly. Second, compared with conventional designed observational studies with multivariable regression models and other statistical analyses, the genetic potency of the filtered instrument variants enables MR to avoid the reverse causal effect. Third, it is the first study implementing MR analysis to focus on the causality between coffee consumption and the infection risk of periodontitis. Considering the high prevalence of periodontitis and plentiful coffee consumption worldwide, revealing the causality between continuous coffee consumption and binary coffee-consuming behavior and infection of periodontitis is instructive, which may affect public health policies for periodontitis prevention and treatment. Fourth, coffee consumption was defined as binary and continuous coffee consumption respectively, bestowing the ability to pertinently concern the behavior and the exact amount and indicating more nuanced conclusions on us.

Nevertheless, there also exist limitations. First, the participants were limited to the European population. The scalability and consistency among other populations remained to be validated. Second, a relatively small amount of SNPs may cause the instability of sensitivity tests, while F-statistics’ deficiency in reverse causality analyses may cause weak instrument bias. Third, the effect scale of causality from continuous coffee consumption on the liability of periodontitis is relatively small, and variant kinds of coffee and decaffeinated coffee and different ingredients should also be focused on, studies concerning the heterogeneity of chronic and acute periodontitis and the validation on the stability of the causality discovered in the article can also be considered in the future.

## Conclusion

The study strengthened the evidence of the causality from continuous coffee consumption to the infection risk of periodontitis, while the association between coffee-consuming behavior and the risk of periodontitis failed to be demonstrated. Given the general treatment for patients infected with periodontitis, intensive attention should be given to a definite amount of coffee consumption instead of focusing on the behavior of consuming coffee, and further studies were required to validate the result and potential modifying effect.

### Supplementary Information

**Additional file 1: Supplementary Table 1.** Characteristics of the SNPs used for analyzing the causality from binary coffee consumption on periodontitis and the result of Mendelian Randomization in IVW, Weighted Median and MR-Egger methods. SNP, single nucleotide polymorphism; EAF, effect allele frequency; SE, standard error; IVW, inverse variance weighted.

**Additional file 2: Supplementary Table 2.** Characteristics of the SNPs used for analyzing the causality from periodontitis on continuous coffee consumption and the result of Mendelian Randomization in IVW, Weighted Median and MR-Egger methods. SNP, single nucleotide polymorphism; EAF, effect allele frequency; SE, standard error; IVW, inverse variance weighted.

**Additional file 3: Supplementary Table 3.** Characteristics of the SNPs used for analyzing the causality from periodontitis on binary coffee consumption and the result of Mendelian Randomization in IVW, Weighted Median and MR-Egger methods. SNP, single nucleotide polymorphism; EAF, effect allele frequency; SE, standard error; IVW, inverse variance weighted.

**Additional file 4: Supplementary Table 4.**

**Additional file 5: Supplementary Table 5.**

**Additional file 6: Supplementary Table 6.**

## Data Availability

Data described in the manuscript, code book, and analytic code will be made available upon request pending application.
