# Supplementary material for: Coffee consumption and periodontitis: a Mendelian Randomization study
Source: Genes Nutr. 2023 Sep 9;18:13. doi: 10.1186/s12263-023-00732-3 (PMC10492363; doi:10.1186/s12263-023-00732-3)
Supplement: Supplementary file 1 — Additional file 1: Supplementary Table 1. Characteristics of the SNPs used for analyzing the causality from binary coffee consumption on periodontitis and the result of Mendelian Randomization in IVW, Weighted Median and MR-Egger methods. SNP, single nucleotide polymorphism; EAF, effect allele frequency; SE, standard error; IVW, inverse variance weighted. [file 12263_2023_732_MOESM1_ESM.docx]

|  | | | Binary coffee consumption (Exposure) | | | | | Periodontitis (Outcome) | | | | | Method | Beta | SE | P-value |
| --- | --- | --- | --- | --- | --- | --- | --- | --- | --- | --- | --- | --- | --- | --- | --- | --- |
| SNP | Effect Allele | Other Allele | EAF | Beta | SE | P-value | Sample size | EAF | Beta | SE | P-value | Sample size |  |  |  |  |
| rs117234665 | A | G | 0.015586 | 0.047688 | 0.010395 | 4.50E-06 | 64949 | NA | -0.0182 | 0.0947 | 0.8477 | 49066 | MR-Egger | 1.1726 | 1.0592 | 0.29 |
| rs11939505 | A | G | 0.161076 | -0.0163 | 0.003448 | 2.30E-06 |  | NA | -0.0134 | 0.0222 | 0.5457 |  |  |  |  |  |
| rs12094804 | G | A | 0.060116 | -0.02437 | 0.005322 | 4.70E-06 |  | NA | 0.0042 | 0.0313 | 0.8934 |  |  |  |  |  |
| rs12785741 | C | G | 0.323954 | -0.01274 | 0.002702 | 2.40E-06 |  | NA | 0.0041 | 0.019 | 0.831 |  |  |  |  |  |
| rs141934938 | A | G | 0.03514 | 0.031454 | 0.006875 | 4.80E-06 |  | NA | -0.0326 | 0.0515 | 0.527 |  |  |  |  |  |
| rs143924958 | G | T | 0.033553 | -0.03451 | 0.007411 | 3.20E-06 |  | NA | -0.2208 | 0.125 | 0.07734 |  |  |  |  |  |
|  |  |  |  |  |  |  |  |  |  |  |  |  | Weighted Median | 0.1371 | 0.4629 | 0.77 |
| rs17701213 | C | T | 0.1079 | -0.02098 | 0.004082 | 2.70E-07 |  | NA | -0.0327 | 0.0322 | 0.3094 |  |  |  |  |  |
| rs1773019 | T | C | 0.030224 | 0.036308 | 0.007381 | 8.70E-07 |  | NA | 0.0603 | 0.0572 | 0.2916 |  |  |  |  |  |
| rs1820987 | T | C | 0.56879 | 0.01172 | 0.002561 | 4.70E-06 |  | NA | 0 | 0.0158 | 0.9992 |  |  |  |  |  |
| rs2065113 | G | A | 0.098808 | -0.02582 | 0.004264 | 1.40E-09 |  | NA | -0.0038 | 0.0253 | 0.8817 |  |  |  |  |  |
| rs2117885 | C | T | 0.186513 | 0.015015 | 0.003246 | 3.70E-06 |  | NA | -0.0192 | 0.0189 | 0.3089 |  |  |  |  |  |
| rs2237526 | T | C | 0.327047 | -0.01295 | 0.002726 | 2.00E-06 |  | NA | 0.0076 | 0.0163 | 0.6419 |  |  |  |  |  |
|  |  |  |  |  |  |  |  |  |  |  |  |  | Inverse Variant Weighted | 0.2210 | 0.3574 | 0.54 |
| rs2472297 | T | C | 0.25771 | 0.017741 | 0.00288 | 7.30E-10 |  | NA | 0.0138 | 0.0204 | 0.4993 |  |  |  |  |  |
| rs62159340 | T | G | 0.192084 | -0.01666 | 0.003248 | 2.90E-07 |  | NA | 0.0177 | 0.0268 | 0.509 |  |  |  |  |  |
| rs6860893 | A | G | 0.477557 | -0.01239 | 0.002532 | 9.90E-07 |  | NA | -0.0033 | 0.0154 | 0.831 |  |  |  |  |  |
| rs6968865 | T | A | 0.626977 | 0.018899 | 0.002589 | 2.90E-13 |  | NA | 0.0228 | 0.0159 | 0.1517 |  |  |  |  |  |
| rs79750331 | A | G | 0.040739 | 0.031939 | 0.006579 | 1.20E-06 |  | NA | 0.0457 | 0.0447 | 0.3068 |  |  |  |  |  |
