# Supplementary material for: Coffee consumption and periodontitis: a Mendelian Randomization study
Source: Genes Nutr. 2023 Sep 9;18:13. doi: 10.1186/s12263-023-00732-3 (PMC10492363; doi:10.1186/s12263-023-00732-3)
Supplement: Supplementary file 3 — Additional file 3: Supplementary Table 3. Characteristics of the SNPs used for analyzing the causality from periodontitis on binary coffee consumption and the result of Mendelian Randomization in IVW, Weighted Median and MR-Egger methods. SNP, single nucleotide polymorphism; EAF, effect allele frequency; SE, standard error; IVW, inverse variance weighted. [file 12263_2023_732_MOESM3_ESM.docx]

|  | | | Periodontitis (Exposure) | | | | | Binary coffee consumption (Outcome) | | | | | Method | Beta | SE | P-value |
| --- | --- | --- | --- | --- | --- | --- | --- | --- | --- | --- | --- | --- | --- | --- | --- | --- |
| SNP | Effect Allele | Other Allele | EAF | Beta | SE | P-value | Sample size | EAF | Beta | SE | P-value | Sample size |  |  |  |  |
| rs10143801 | A | G | NA | -0.084 | 0.0171 | 8.66E-07 | 49066 | 0.7281 | 0.0020 | 0.0018 | 0.28 | 428860 | MR-Egger | 0.0016 | 0.0058 | 0.80 |
| rs138868497 | T | C | NA | 1.6387 | 0.3324 | 8.20E-07 |  | 0.9919 | 0.0052 | 0.0095 | 0.58 |  |  |  |  |  |
| rs151226594 | T | G | NA | -0.3671 | 0.0768 | 1.75E-06 |  | 0.9837 | 0.0032 | 0.0065 | 0.63 |  | Weighted Median | -0.0031 | 0.0055 | 0.58 |
| rs73155039 | A | G | NA | 0.8316 | 0.1757 | 2.22E-06 |  | 0.9848 | -0.0077 | 0.0070 | 0.27 |  |  |  |  |  |
| rs76734229 | A | G | NA | -0.1761 | 0.037 | 1.94E-06 |  | 0.0899 | -0.0039 | 0.0028 | 0.17 |  | Inverse Variant Weighted | -0.0016 | 0.0048 | 0.73 |
| rs9954920 | C | G | NA | 0.0769 | 0.0163 | 5.44E-07 |  | 0.3842 | -0.0021 | 0.0017 | 0.19 |  |  |  |  |  |
