# Supplementary material for: Coffee consumption and periodontitis: a Mendelian Randomization study
Source: Genes Nutr. 2023 Sep 9;18:13. doi: 10.1186/s12263-023-00732-3 (PMC10492363; doi:10.1186/s12263-023-00732-3)

| SNP | Effect Allele | Other Allele | Continuous coffee consumption (Exposure) | | | | | Periodontitis (Outcome) | | | | | Method | OR (95%CI) | P-value |
| --- | --- | --- | --- | --- | --- | --- | --- | --- | --- | --- | --- | --- | --- | --- | --- |
|  |  |  | EAF | Beta | SE | P-value | Sample size | EAF | Beta | SE | P-value | Sample size |  |  |  |
| rs574367 | T | G | 0.21 | 1.025 | 0.424 | 8.06E-9 | 375833 | NA | 0.0284 | 0.0194 | 0.14 | 49066 | MR-Egger | 1.0002 | 0.99 |
| rs1260326 | C | T | 0.61 | 1.166 | 0.387 | 2.62E-19 |  | NA | 5E-4 | 0.0157 | 0.97 |  |  |  |  |
| rs4410790 | C | T | 0.63 | 1.985 | 0.387 | 5.59E-141 |  | NA | 0.0214 | 0.0159 | 0.18 |  |  |  |  |
| rs73073176 | C | T | 0.87 | 1.520 | 0.469 | 5.56E-25 |  | NA | 0.0246 | 0.0253 | 0.33 |  | Inverse Variance Weighted | 1.0078 | 0.06 |
| rs34060476 | G | A | 0.13 | 1.375 | 0.469 | 5.06E-18 |  | NA | -0.0224 | 0.0249 | 0.37 |  |  |  |  |
| rs1057868 | T | C | 0.29 | 1.404 | 0.4 | 5.26E-33 |  | NA | 0.0063 | 0.017 | 0.71 |  |  |  |  |
| rs1956218 | G | A | 0.56 | 0.906 | 0.387 | 3.62E-8 |  | NA | 0.0198 | 0.0157 | 0.21 |  |  |  |  |
| rs2472297 | T | C | 0.27 | 2.131 | 0.412 | 5.19E-155 |  | NA | 0.0138 | 0.0204 | 0.50 |  | Weighted Median | 1.0070 | 0.19 |
| rs66723169 | A | C | 0.23 | 1.212 | 0.424 | 9.88E-17 |  | NA | 0.0087 | 0.0195 | 0.66 |  |  |  |  |
| rs2330783 | G | T | 0.99 | 2.128 | 0.794 | 1.57E-12 |  | NA | -0.0343 | 0.0639 | 0.59 |  |  |  |  |

P for Cochran’s Q test: 0.79 for MR-Egger, 0.84 for IVW;

P for MR-Egger intercept test: 0.52

P for MR-PRESSO: 0.88

rs2330783 was removed for its strong correlation with alcohol consumption and hyperlipidemia.


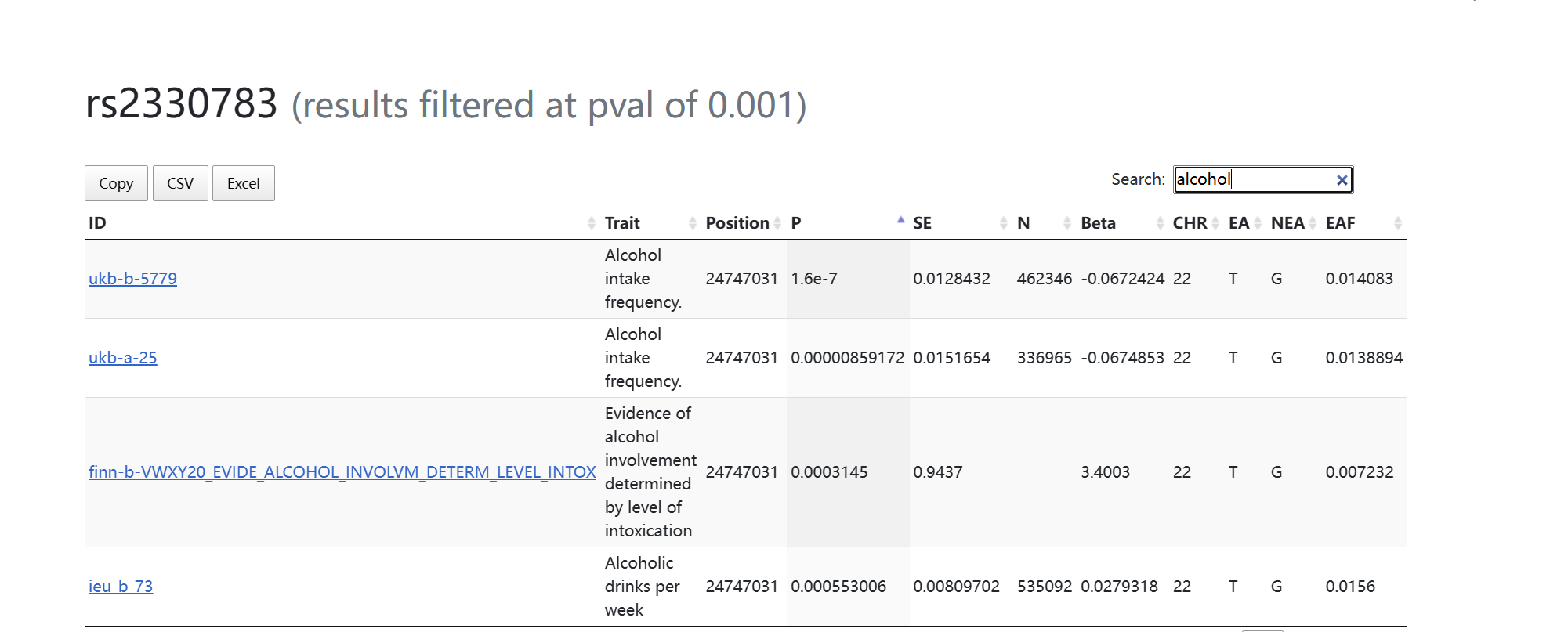


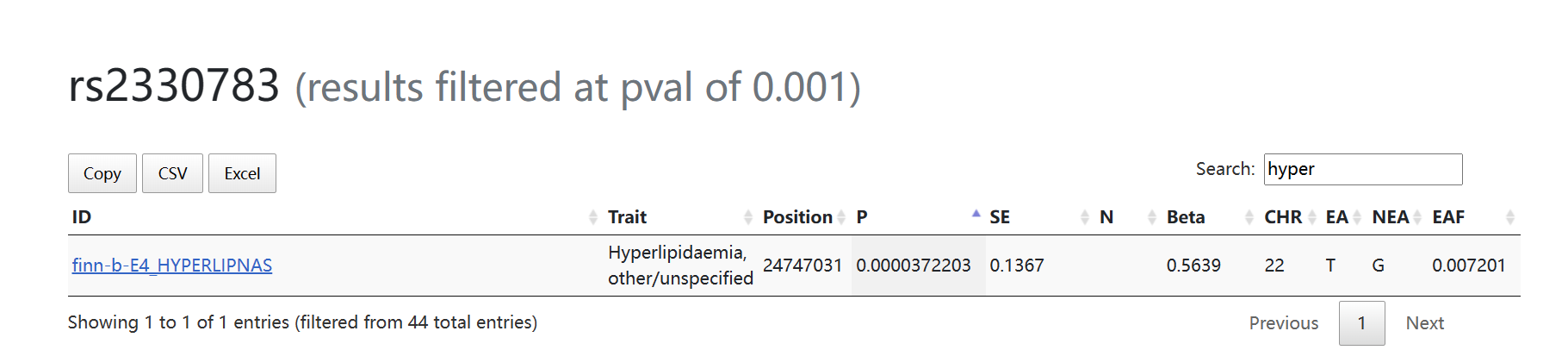


rs34060476 was removed for its strong correlation with diabetes mellitus and hyperlipidemia.


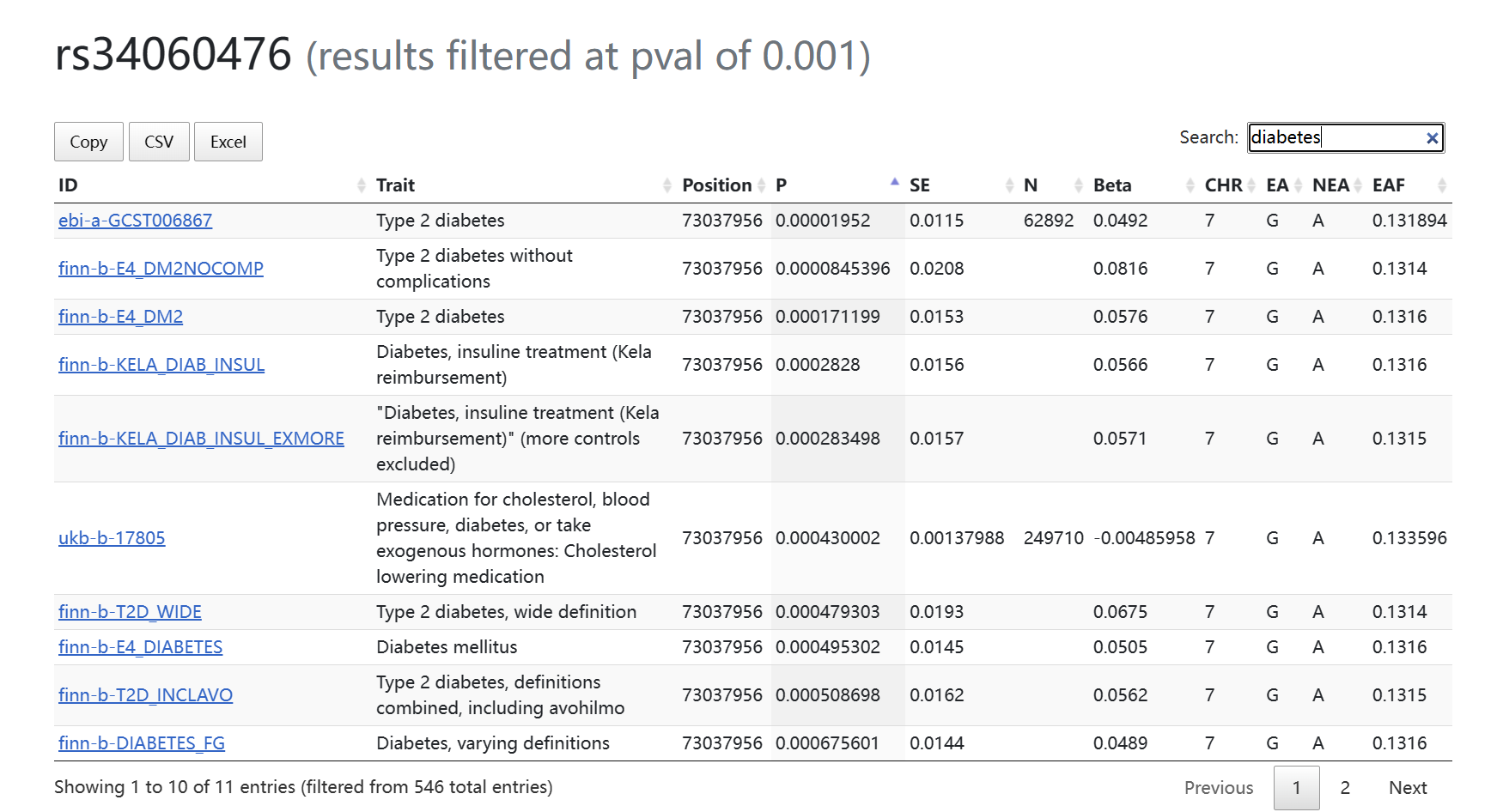


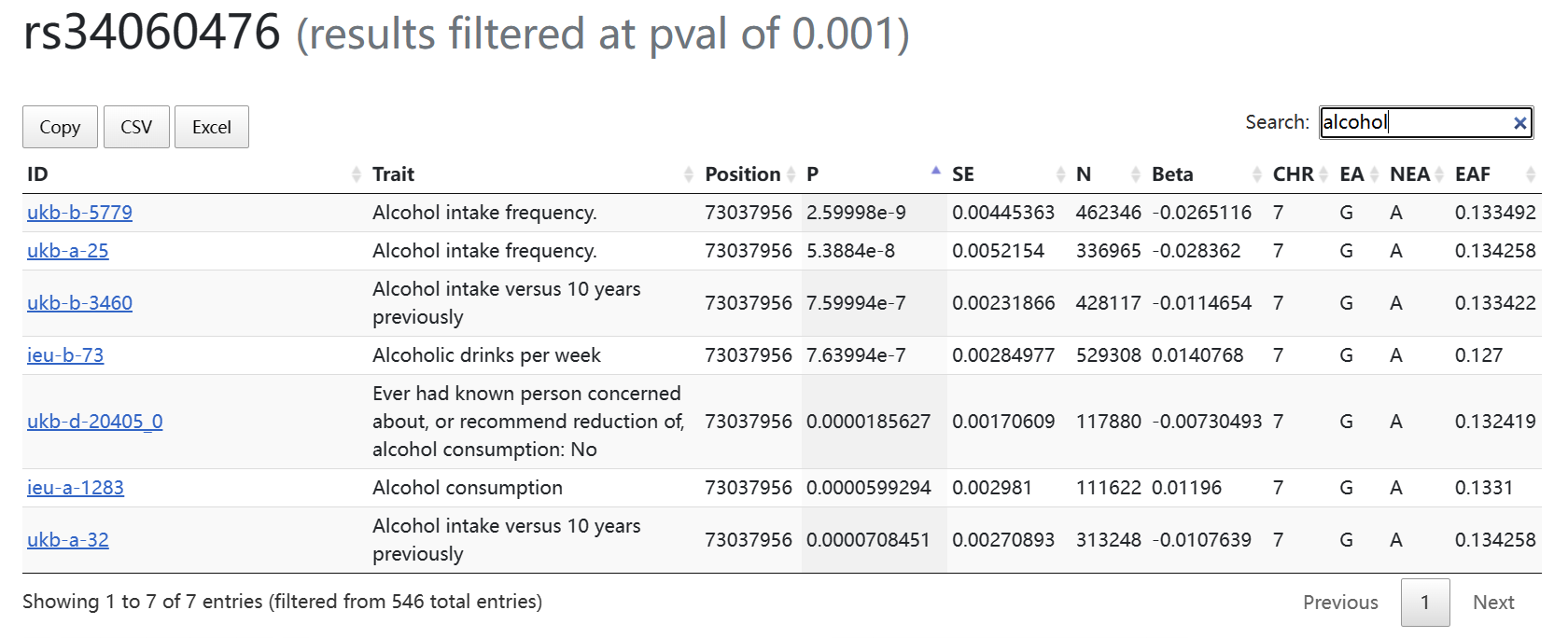

Supplement: Supplementary file 4 — Additional file 4: Supplementary Table 4. [file 12263_2023_732_MOESM4_ESM.docx]
